# Upper-Body Post-activation Performance Enhancement for Athletic Performance: A Systematic Review with Meta-analysis and Recommendations for Future Research

**DOI:** 10.1007/s40279-021-01598-4

**Published:** 2021-11-26

**Authors:** Mitchell James Finlay, Craig Alan Bridge, Matt Greig, Richard Michael Page

**Affiliations:** Sports Injuries Research Group, Department of Sport and Physical Activity, Edge Hill University, St. Helens Road, Ormskirk, L39 4QP Lancashire UK

## Abstract

**Background:**

Research on post-activation performance enhancement (PAPE) is dominated by lower-body conditioning activities/performance test complexes. Despite the contribution of the upper body to many sporting actions, no review on upper-body PAPE currently exists.

**Objectives:**

The aim of this systematic review with meta-analysis was to provide a synthesis of the available research on the inclusion of upper-body PAPE conditioning activities to improve athletic performance.

**Methods:**

A review of the literature was conducted according to the Preferred Reporting Items for Systematic Review and Meta-analyses guidelines, including a literature search of EBSCOhost, SPORTDiscus, PubMed and Google Scholar databases. A total of 127 studies were identified through database searches, and were assessed against the following criteria: (1) randomised controlled trial or pre-and-post study design; (2) studies explored the effects of prior voluntary muscle activity, and not electrically induced contractions, (3) evidence, or lack thereof, of PAPE was quantified by the monitoring of individual performance to commonly applied physical tests or sport-specific tasks; (4) conditioning activities and performance tests were primarily upper-body; (5) detailed description of a standardised warm-up; and (6) full-text versions of studies could be accessed in English language peer-reviewed journals. Studies were quality assessed for methodological quality via the PEDro scale and ranked accordingly.

**Results:**

Thirty-one studies met the inclusion criteria. Studies were classified into different conditioning activity modes: bench press variations, sport-specific (modified implement throws, swing-specific, cable pulley, elastic resistance, combination) and bodyweight activity. Acute performance enhancement in several movement-specific combinations was found. A meta-analysis revealed that bench press at  ≥ 80% one repetition maximum significantly (*p* = 0.03; ES = 0.31) improves subsequent power output in the ballistic bench throw at 30–40% one repetition maximum, following 8–12 min recovery. Additionally, sport-specific overweight implement throws improved subsequent throwing distance at competition weight by ~ 1.7–8.5%; ES = 0.14–0.33, following 3 min recovery. Sport-specific lighter weighted bat swings and swing-specific isometrics resulted in improved subsequent competition weight bat swing velocities, ranging from ~ 1.3–4.9%; ES = 0.16–0.57.

**Conclusions:**

This review presents several upper-body movement-specific conditioning activities that could be considered by coaches and practitioners as part of complex or contrast training, or used in pre-competition warm-ups to acutely enhance performance.

## Key Points

**Table:** 

Bench press of ≥ 80%1RM induces a moderate post-activation performance enhancement effect in the subsequent ballistic bench throw.
Sport-specific post-activation performance enhancement conditioning activities, including overweight implement throw and lightweight and isometric bat swings, improved subsequent throwing distance and bat swing velocity, respectively.
Upper-body conditioning activities that share biomechanical specificity with the performance test may be more likely to induce a post-activation performance enhancement effect.

## Introduction

Coaches and practitioners regularly utilise the pre-competition warm-up to acutely enhance neuromuscular performance [1–3]. The use of a warm-up is thought to influence performance through several temperature-related (decreased resistance of muscles and joints, increased nerve conduction rate and thermoregulatory strain, greater release of oxygen from haemoglobin and myoglobin, and speeding up of metabolic reactions) and non-temperature-related (increased blood flow, elevation of baseline oxygen consumption and psychological effects) mechanisms [3]. Likewise, it is possible that the use of an additional conditioning activity may also influence subsequent neuromuscular performance [4], possibly above and beyond that of the warm-up [5]. An example use of a conditioning activity, is complex training, whereby the completion of a high-load resistance activity can enhance subsequent plyometric or ballistic-type activity [4, 6, 7]. The mechanisms and terminology of this acute exercise performance enhancement, or previously termed ‘post-activation potentiation’ (PAP), have recently been subject to debate within the scientific community, resulting in the term ‘post-activation performance enhancement’ (PAPE) being proposed [8]. Blazevich and Babault [5] suggest that PAPE may be attributable to mechanisms that are commonly observed through warm-ups, such as changes in muscle temperature and intramuscular fluid accumulation, or neural mechanisms. However, the specific mechanisms require further investigation. A delayed, yet prolonged window of action is associated with PAPE, and is often observable by improvements in neuromuscular performance lasting several minutes in responding athletes [5, 9, 10]. Where PAP may also occur and coexist with PAPE in response to prior voluntary action [9], it is typically dissipated in a manner of minutes, often by the time PAPE effects are evident [5]. With consideration to the traditional misuse of PAP, and the ongoing debates on revised nomenclature [5, 9, 11, 12], this current review considers studies that have assessed the efficacy of prior voluntary muscular activity, on the subsequent performance of a voluntary activity, as PAPE.

Considerable inter-individual variability exists in the PAPE response, from a positive effect (responders), no effect (non-responders) or even adverse effects [13]. There appear to be several modulating factors including, but not limited to, participant strength levels, sex, conditioning activity, type of load, warm-up activity, rest-period and performance test [4, 5]. The warm-up is perhaps of increased importance, in that it could also explain any performance enhancement as opposed to, or in parallel with the conditioning activity, considering that the proposed mechanisms are similar. Nevertheless, recent reviews related to PAPE show that numerous forms of prior voluntary activity can improve subsequent neuromuscular performance, such as barbell compound lifts, plyometric, ballistic, variable resistance, resisted sprints and isometric activity [4, 14, 15]. These reviews have predominantly focussed on lower-body PAPE conditioning activities and performance tests [4, 7, 14] because of a paucity of research pertaining to the upper-body, perhaps with the exception of bench press variations [14]. The recent increase in experimental research on upper-body PAPE means it is now conceivable to perform a more focused and up-to-date systematic review and meta-analysis in this area.

Seitz and Haff [4] reported small and moderate PAPE effects for jumping (effect size [ES] = 0.31) and sprinting (ES = 0.50) performance, in addition to small effects for throwing (ES = 0.28) and upper-body ballistic performance (ES = 0.23). Whilst this suggests upper-body performance can be aided by PAPE, important detail on the different types and characteristics of individual upper-body PAPE conditioning activities was often lacking. Furthermore, there may be a need for a more up-to-date review inclusive of more recent PAPE research in the literature. Considering the above reasons, a more comprehensive and recent review of literature on upper-body complexes is required.

Therefore, this study aims to conduct a systematic review on the current evidence of upper-body PAPE conditioning activities. To do this, a systematic process of literature searching, data extraction and quality assessment is conducted. Data from relevant studies are collated and shown as percentage changes and ES where possible. Finally, where appropriate, a meta-analysis of similar study designs will be performed. Considering the importance of upper-body strength and power to many sports [16], a more comprehensive and up-to-date review on upper-body PAPE could be valuable for the coach and practitioner in assisting the selection and use of acute strategies to facilitate performance for short-term, medium-term and long-term goals.

## Methods

### Literature Search

**Fig. 1:**
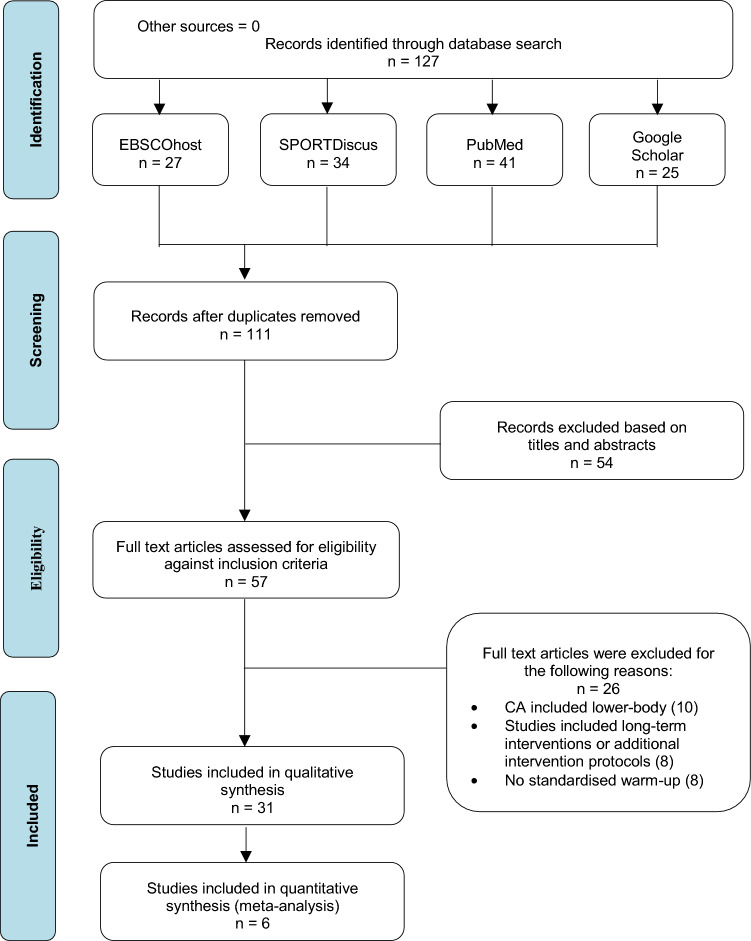
Preferred Reporting Items for Systematic Reviews and Meta-Analysis (PRISMA) flow diagram of the literature screening process. *CA* conditioning activity

A review of the literature was conducted according to the Preferred Reporting Items for Systematic Review and Meta-analyses (PRISMA) guidelines [17] (Fig. 1). EBSCOhost, SPORTDiscus, PubMed and Google Scholar databases were searched until August 2021 for all studies pertaining to acute upper-body PAPE. Several combinations of search terms detailed in Table 1 were used. Reference lists of the identified manuscripts were searched manually.

**Table 1:** Search criteria

Search terms	
“Post-activation potentiation” OR “PAP” OR “post-activation performance enhancement” OR “PAPE” OR “warm-up” OR “contrast” OR “complex” OR “acute” OR “acutely” OR “effects” OR “short-term” OR “preparedness” OR “pre-activation” OR “activation” OR “resisted warm-up” OR “pre-competition” OR “neuromuscular” OR “strength” OR “power” AND “Upper-body” OR “throwing” OR “ballistic” OR “striking” OR “bench press” OR “dry-land” OR “exercise” OR “series” OR “bout” OR “athlete” OR “athletic” OR “sport”	

### Data Extraction

The screening and data extraction process was carried out by two independent reviewers (MF and RP) and included study results, sample size, sex, experience level and strength levels. Any discrepancies between the two reviewers were discussed between the authors until a consensus was reached. Studies were assessed for methodological quality at the study level using the Physiotherapy Evidence Database (PEDro) scale [18]. Total PEDro scores are reached based on satisfaction of criterion measures relating to participant allocation, allocation concealment, blinding of participants, therapists and assessors, and the provision of sufficient statistical information [18]. A total of 11 criterion measures are assessed; however, criterion measures 1 and 6 are to assess external validity and blinding of therapists who administered the therapy, and are not included in the total PEDro score. Therefore, a total score of 9 is attainable.

### Study Identification and Selection

For inclusion in the review, the following criteria were required to be satisfied: (1) randomised controlled trial or pre-and-post study design; (2) studies explored the effects of prior voluntary muscle activity; (3) evidence, or lack thereof, of PAPE was quantified by the monitoring of individual performance to commonly applied physical tests or sport-specific tasks; (4) conditioning activities and performance tests were primarily upper body; (5) studies included a detailed description of an appropriate, standardised warm-up; (6) full-text versions of studies could be accessed in English language, in peer-reviewed journals. Studies were excluded from the review if they included any of the following criteria: (1) a conditioning activity or performance measure that was primarily lower body, (2) long-term interventions or considered other interventions, such as nutritional supplementation; and (3) no details of a standardised warm-up.

### Coding of the Studies

The same two authors that performed the data extraction (MF and RP) performed the study coding. Specifically, studies that passed eligibility criteria were classified into groups according to the conditioning activity performed, which included bench press variations, sport-specific, bodyweight and combined activity. The bench press variation category included studies that explored the bench press and eccentric, concentric, isometric and variable resistance variations of the bench press. The sport-specific category included studies that investigated the use of a conditioning activity that replicated the sporting task. This included modified implement throws, swing-specific activity in ball striking sports, cable pulley and elastic resistance conditioning activities. Studies that included only bodyweight activity, or included a combination of exercises, were classified as such.

### Risk of Bias Analysis

Articles that passed the eligibility criteria were ranked on their methodological quality via the PEDro scale [18]. This comprised the binary scoring (0/1) of whether articles followed 9/11 discrete criteria, Table 2. Criterion 1 was concerned with assessing external validity; therefore, it was deemed not applicable for this review. Similarly, criterion 6 was omitted. Studies were subsequently scored on the remaining nine criteria.

**Table 2:** PEDro scale quality assessment of the articles [21]

Reference	1^a^	2	3	4	5	6^a^	7	8	9	10	11	Total
Ulrich and Parstorfer [25]	–	1	0	0	0	–	0	1	1	1	1	5/9
Judge et al. [26]	–	1	0	0	0	–	0	1	1	1	1	5/9
West et al. [27]	–	0	0	0	0	–	0	1	1	1	1	4/9
Tsolakis et al. [28]	–	1	0	1	0	–	0	1	1	1	1	6/9
Ferreira et al. [29]	–	1	0	0	0	–	0	1	1	1	1	5/9
Abbes et al. [30]	–	1	0	1	0	–	0	1	1	1	1	6/9
Krzysztofik [31]	–	1	0	1	0	–	0	1	1	1	1	6/9
Krzysztofik and Wilk [32]	–	1	0	1	0	–	0	1	1	1	1	6/9
Markovic et al. [33]	–	1	0	1	0	–	0	1	1	1	1	6/9
Brandenburg [34]	–	1	0	1	0	–	0	1	1	1	1	6/9
Esformes et al. [35]	–	1	0	1	0	–	0	1	1	1	1	6/9
Montoya et al. [36]	–	1	0	0	0	–	0	1	1	1	1	5/9
Bellar et al. [37]	–	1	0	0	0	–	0	1	1	1	1	5/9
Bevan et al. [38]	–	0	0	0	0	–	0	1	1	1	1	4/9
Bodden et al. [39]	–	1	0	1	0	–	0	1	1	1	1	6/9
Kilduff et al. [40]	–	0	0	0	0	–	0	1	1	1	1	4/9
Liossis et al. [41]	–	1	0	0	0	–	0	1	1	1	1	5/9
Judge et al. [42]	–	1	0	0	0	–	0	1	1	1	1	5/9
Gilmore et al. [43]	–	0	0	0	0	–	0	1	1	1	1	4/9
Hancock et al. [44]	–	1	0	1	0	–	0	1	1	1	1	6/9
Barbosa et al. [45]	–	1	0	1	0	–	0	1	1	1	1	6/9
Martinez-Garcia et al. [46]	–	0	0	1	0	–	0	1	1	1	1	5/9
Gelen et al. [47]	–	1	0	1	0	–	0	1	1	1	1	6/9
Cuenca-Fernandez et al. [48]	–	1	0	1	0	–	0	1	1	1	1	6/9
Bliss et al. [49]	–	0	0	1	0	–	0	1	1	1	1	5/9
Feros. 2020 [50]	–	1	0	0	0	–	0	1	1	1	1	5/9
Higuchi et al. [51]	–	0	0	1	0	–	0	1	1	1	1	5/9
Williams et al. [52]	–	1	0	1	0	–	0	1	1	1	1	6/9
Sarramian et al. [53]	–	0	0	0	0	–	0	1	1	1	1	4/9
Smilios et al. [54]	–	1	0	1	0	–	0	1	1	1	1	6/9
Asencio et al. [55]	–	1	0	0	0	–	0	1	1	1	1	5/9

1 = criterion was satisfied, 0 = criterion was not satisfied. Each satisfied criterion measure, excluding item 1 and 6, contributes 1 point to the total PEDro score (1–9). Criteria: (1) eligibility criteria were specified (^a^not applicable); (2) random allocation; (3) concealed allocation; (4) groups similar at baseline; (5) blinding of participants; (6) blinding of therapists who administered the therapy (^a^not applicable); (7) blinding of assessors; (8) less than 15% drop-outs; (9) intention to treat; (10) between-group statistical analysis; (11) point measures and variability data

*PEDro* Physiotherapy Evidence Database

### Reporting of Results

Results from the included studies are presented in the main text and in Table 3 as percentage changes. Effect sizes were also calculated, according to a previous review relating to PAPE [4] and more recently [19]. Specifically, the ES were calculated using Hedges and Olkin’s *g* (Hedges *g)* [20] as follows (Eq. 1):

1 $${\text{ES}} = {\text{g }}\frac{{\left( {M_{{{\text{post}}}} - M_{{{\text{pre}}}} } \right)}}{{{\text{SD}}_{{{\text{pooled}}}} }},$$

where *M*_post_ is the mean of the performance test completed after the conditioning activity, *M*_pre_ is the mean of the performance test completed before the CA and *SD*_pooled_ is the pooled standard deviation of the measurements (Eq. 2):

2 $${\text{SD}}_{{{\text{pooled}}}} = \frac{{\left( {\left( {{\text{n}}_{1} - 1} \right){ } \times {\text{SD}}{}_{1}^{2} + ({\text{n}}_{2} - 1} \right){ } \times {\text{SD}}{}_{2}^{2} ) }}{{({\text{n}}_{1} + {\text{n}}_{2 } - { }2)}},$$

where ${\text{SD}}_{1}^{2}$ is the standard deviation of the performance test completed before the CA and ${\text{SD}}_{2}^{2}$ is the standard deviation of the performance test completed after the conditioning activity. Hedges and Olkin [20] suggest the absolute value of the ES is over-estimated where there are small sample sizes. The authors advise that the ES should be corrected (Hedges g*) as using the following equation (Eq. 3):

3 $${\text{Correction factor}} = 1 - { }\frac{3}{{4({\text{n}}_{1} + {\text{ n}}_{2 } - { }2){ } - { }1}}.$$

**Table 3:** Studies investigating upper-body PAPE

Reference	Participants	Familiarisation	Warm-up	Time from warm-up to CA	CA sets × reps and rest between sets	Control	Performance measure	Time course post-CA	Results	*g*
Bench press variation CA										
Bevan et al. [38]	26 M pro rugby players BP1RM = 1.35 × BM Age 26 ± 5 y	Yes	5 min rowing ergometer, DS of associated musculature	U	3 × 3 BP at 87%1RM 4 min	No	BBT (40%1RM)	15 s, 4, 8, 12, 16, 20, 24 min	~ 5.4% increase in throw height at 8 min post-BP	1.34
									~ 4.2% increase in PPO at 8 min post-BP	0.34
Brandenburg [34]	8 M resistance-trained individuals BP1RM = 1.4 × BM Age 24.4 ± 4 y	Yes	5 min low-intensity cycling, 2 × 10 sets of BBT at 40–50%1RM	4 min	1 × 5 BBT at varied load 50–100%1RM	Yes	BBT (40%1RM)	4 min	No significant improvements in mean PO at all loads	-
Liossis et al. [41]	9 U amateur combat athletes BP1RM = 1.1 – BM Age 26.1 ± 3.4 y	Yes	5 min low-intensity jogging, 4 sets of incremental BP at 30–50%1RM	U	1 × 5 BP at 65–85%1RM	No	BBT (30%1RM)	4, 8 min	~ 5% increase in PPO in 65%1RM BP after 4 min rest	0.35
									~ 3.8% increase in PPO in 85%1RM BP after 8 min rest	0.33
West et al. [27]	20 M pro rugby players BP1RM = 1.48 × BM Age 26.5 ± 4.1 y	Yes	5 min rowing ergometer, DS of upper body	10 min	3 × 3 BBT at 30%1RM OR 3 × 3 BP 87%1RM Individual rest times	No	BBT (30%1RM)	8 min	~ 4.3% increase in PPO post-BP	0.34
									~ 3.6% increase in PPO post-BBT	0.28
Esformes et al. [35]	10 M competitive rugby players BP1RM = ~ 1.1 × BM Age 20.4 ± 0.8 y	Yes	5 min low-intensity cycling, DS of upper body	15 min	1 × 3 BP OR ECC BP OR CONC BP at 3RM OR 1 × 7 s ISO BP	No	BBT (40%1RM)	12 min	~ 3.3% increase in PPO post-CONC BP	0.15
									~ 2.8% increase in PPO post-ISO BP	0.14
									~ 0.8% increase in PPO post-ECC BP	0.06
									~ 0.5% decrease in PPO post-BP	-
Kilduff et al. [40]	27 M pro rugby players BP1RM = 1.27 × BM Age 24 ± 3.4 y	Yes	5 min low-intensity cycling, DS of upper body	10 min	1 × 3RM BP	No	BBT (40%1RM)	~ 15 s, 4, 8, 12, 16, 20 min	− 4.7% decrease in PPO after ~ 15 s rest	-
									~ 2.8% increase in PPO after 8 min rest	0.19
									~ 5.3% increase in PPO after 12 min rest	0.35
Krzysztofik et al. [31]	12 M resistance-trained individuals BP1RM = 1.4 × BM Age 25.2 ± 2.1 y	Yes	5 min low-intensity cycling, DS of upper body, 10 push-ups	5 min	3 × 3 BP at 85%1RM 4 min	Yes	BP to failure (60%1RM)	4, 8, 12, 16 min	No significant differences in reps, PO or velocity	-
Tsolakis et al. [28]	23 (13 M 10 F) Elite fencers M BP1RM = 1.01 × BM F BP1RM = 0.62 × BM Age 22.25 ± 4.25 y	Yes	5 min light jogging, 15 s whole body static stretch holds, incremental BP (50–75%1RM)	3 min	3 × 3 s ISO BP 15 s	No	BBT (40%1RM)	15 s, 4, 8, 12 min	No significant differences	-
Smilios et al. [54]	11 M volleyball players BP1RM = 0.94 × BM Age 16.5 ± 0.5 y	No	Stretching of upper body, 10 push-ups, 2 × 5 BP at 60–80% of exercise load	3–7 min	1 × 3 BBT at 30%1RM OR 1 × 3 BBT at 30%1RM + 1 × 5 BBT at 60%1RM	Yes	BBT (30%)	3, 5 min	~ 8.7% increase in average PO in contrast compared with BBT only after 3 min rest	0.51
									~ 10.4% increase in mechanical PO in contrast compared with BBT after 5 min rest	0.60
Ulrich and Parstorfer [25]	16 M recreational athletes BP1RM = 1.18 × BM Age 23.1 ± 3.2 y	No	5 min cycling (< 100 W at 60–80 rpm), DS of upper body	8 min	1 × 3 BP at 80%1RM OR 1 × 3 ECC BP at 120%1RM	No	BBT (30%)	1, 4, 8, 12, 16 min	~ 5.7% increase in PPO post-BP after 8 min rest No significant increases in PPO post-ECC	0.37
Asencio et al. [55]	14 M amateur handball players BP1RM = 1.08 × BM Age 23.76 ± 3.72 y	Yes	Light jogging, DS, core exercises, 10 push-ups, 10 handball passes and throws	U	1 × 3 BP at 90%1RM	No	Handball throw velocity	4 min	No significant improvements	-
Ferreira et al. [29]	11 M resistance- trained individuals BP1RM = 1.03 × BM Age 25 ± 4 y	Yes	5 min run on treadmill, lower-body stretching, incremental BP at 50–70%1RM	3 min	1 × BP1RM protocol	No	CONC BP (50%1RM)	1, 3, 5, 7 min	Significant increase (*p* < 0.05) in PPO post-BP after 7 min	–
Markovic et al. [33]	23 M physically active individuals BP1RM = 1.01 × BM Age 22.0 ± 3.1 y	Yes	5 min low-intensity jogging, calisthenics, 15 sit-ups, back extensions and push-ups. Light stretching	15 min	1 × 6 BP at 60%1RM 2 × BP 3RM 3 min	Yes	Seated MB throw velocity (0.55 or 4 kg)	3 min	~ 8.3% increase in throwing velocity of 4-kg ball post-BP trial	–
									No significant difference in throwing velocity of 0.55 kg MB post-BP trial	–
Bodden et al. [39]	14 M resistance- trained individuals BP1RM = 1.3 × BM Age 21 ± 1.5 y	Yes	25 jumping jacks, 10 forward/backward circles, shoulder taps and push-ups	1 min	CONC BP OR CONC BBT 1 × 3 at 30%, 1 × 3 at 50% 1 × 3 at 70% and 1 × 2 at 90% of BP1RM 1–3 min	Yes	PLYO Push-up	1 min	No improvements in net impulse and take-off velocity in CONC BP and CONC BBT, compared to CONT	-
Martinez-Garcia et al. [46]	14 F experienced handball players	No	5 min jogging, 5 min ballistic movements and overhead throw gestures	U	1 × 5 unilateral standing chest press with VR OR	No	Overhead handball throw velocity	< 1, 1, 2, 10 min	No significant improvements post-VR	-
	Age 21.2 ± 2.7 y				1 × 5 unilateral standing ISO chest press				No significant improvements post-ISO	-
Sport-specific CA										
Modified implement throw CA										
Judge et al. [42]	41 (23 M 18 F) college throwers RTH 4.7 ± 0.93 Age 18–24 y	Yes	15 min general WU; skipping, dynamic mobility	U	OHB at (standard) OR + 1 kg (heavy) OR − 1 kg (light)	Yes	OHB distance at competition load	3 min	~ 1.7% increase in mean distance after heavier trial compared with standard trial	0.14
Bellar et al. [37]	17 (9 M 8 F) elite college throwers PC1RM = 1.11 × BM Age 22.9 ± 3.15 y	No	15 min general WU; skipping, dynamic mobility	U	1 × 5 weight throws at (standard) OR + 1.37 kg (heavy) OR + 2.27 kg (heavier) 3 min	Yes	Weight throw distance at competition load	3 min	~ 3.2% increase in mean distance after 1.37 kg heavier trial compared with standard trial	0.19
									~ 3.2% increase in mean distance after 2.27 kg heavier trial compared with standard trial	0.19
Judge et al. [26]	10 U (15.9 ± 1.2 y) throwing athletes PC1RM = 0.87 × BM Age 15.9 ± 1.15 y	No	15 min general WU; drills, skipping, dynamic mobility	U	Weight throw at (standard) OR + 1.37 kg OR + 2.27 kg 3 min	Yes	Weight throw distance at competition load	3 min	~ 8.5% increase in mean distance after 1.37 kg heavier trial compared with CONT	0.33
									~ 6.3% increase in mean distance after 2.27 kg heavier trial, compared with CONT	0.26
Feros [50]	13 M amateur cricket bowlers	No	20-m shuttle runs at 50%, walking lunges, skipping, progressive effort sprints, whole body DYNWU, cricket bowls at varied intensities	3 min	1 × 6 cricket bowls at (standard) OR + 10% heavier OR − 10% lighter	Yes	Cricket bowling speed and accuracy	3 min	No significant improvements in ball speed or accuracy post heavier or lighter, compared with control	-
Swing-specific CA in ball striking sports										
Gilmore et al. [43]	28 F softball players BP1RM = 0.63 × BM Age 20 ± 2.6 y	No	2 min jogging on a treadmill at 6 mph, DS. 1 × 5 dry-swings, 1 × 5 WU swings	10–15 min	3 × 5 s swing-specific ISO 30 s	No	Softball swing velocity	1, 2, 4, 6, 8, 10, 12 min	~ 4.9% increase in bat swing velocity 6 min post-ISO	0.44
Higuchi et al. [51]	24 M collegiate baseball players	No	Jogging, stretching, calisthenics, practice swings until participants ‘felt ready’	1–2 min	4 × 5 s swing-specific ISO 5 s. OR 1 × 5 SWU 5 s. OR 1 × 5 WBS (~ + 0.68 g) 5 s	Yes	Baseball swing velocity	1 min	~ 1.3% increase in bat swing velocity following ISO	0.16
									~ 1.1% decrease in bat swing velocity post-SWU	-
									~ 2.9% decrease in bat swing velocity post-WBS	-
Montoya et al. [36]	19 M recreational baseball players Age 24.5 ± 3.9 y	Yes	3 min upper-body ergometer at 50 rpm, practice swings	U	1 × 5 standard load bat swings at (0.89 kg standard) OR, + 0.272 kg (light) OR 1.56 kg (heavy) 5 s	Yes	Baseball swing velocity	30 s	~ 3.3% increase in bat swing velocity after light trial compared with standard	0.57
									~ 4.6% decrease in bat swing velocity after heavy trial compared with standard	-
Williams et al. [52]	15 U NCAA D1 baseball players Age 19.93 ± 1.27 y	No	25 jumping jacks. 10 BW squats, 10 walking knee-hugs, 10 straight leg marches, 10 push-ups	U	1 × 5 SB OR + 10.6 oz fungo OR + 25.6 oz weighted gloves OR + 25.6 oz donut	Yes	Baseball swing maximal resultant velocity and resultant velocity at ball contact	1 min, then 1 swing every 20 s (1 × 5)	No significant differences between all warm-up implements	-
Bliss et al. [49]	13 M skilled golfers Age 20 ± 1 y	No	DS; 2 min skipping, 1 × 10 leg swings, 2 × 10 ER shoulder external rotations, 1 × 10 kneeling kickbacks, 1 × 10 lunges with rotations, 1 × 12 OH squat, golf-specific shots	U	SS swings with; light dominant side × 10, light non-dominant side × 10, medium dominant side × 10, heavy dominant side × 10 60 s	Yes	Clubhead and ball speed, carry distance and total distance	1 min. Then 1 drive per minute for 9 min	~ 1.4% and 0.9% increase in clubhead and ball speed, respectively post-SS compared with CONT	0.27 0.17
									~ 2.6% and 1.4% increase in carry and total distance post-SS compared with CONT	0.40 0.22
Cable pulley CA										
Hancock et al. [44]	30 (15 M 15 F) college-level swimmers Age 20.1 ± 1 y	No	900-m SWIMWU	Immediately after	4 × 7 s resisted CP swim sprints at individual loads 1 min	Yes	100-m freestyle swim time	6 min	~ 0.9% decrease (0.54 s) in 100-m swim time following resisted sprints trial, compared with CONT	0.10
Asencio et al. [55]	14 M amateur handball players BP1RM = 1.08 × BM Age 23.76 ± 3.72 y	Yes	Light jogging, DS, core exercises, 10 push-ups, 10 handball passes and throws	U	1 × 6 CP ECC	No	Handball throw velocity	4 min	No significant improvements	-
Cuenca-Fernandez et al. [48]	20 M national-level swimmers Age 18.0 ± 1.39	Yes	SWIMWU 400 m varied swim technique and paces	U	SWIMWU + DRYWU; dynamic stretching and 1 × 3 CP overs at 85% of 1RM OR SWIMWU CONT	Yes	Semi-tethered resisted front crawl 15-m swim	6 min	~ 9.4% increase in rate of force development following + DRYWU compared with SWIMWU	0.21
									5.1% increase in stroke rate following + DRYWU compared with SWIMWU	0.36
									~ 13.7% and ~ 15% decrease in velocity and power following + DRYWU compared with SWIMWU	-
									~ 2.6% and ~ 30.4% decrease in force and acceleration following + DRYWU compared with SWIMWU	-
Elastic resistance CA										
Barbosa et al. [45]	12 M national-level and international-level swimmers Age 23.5 ± 3.6 y	Yes	SWIMWU 700 m varied techniques OR CONT SWIMWU 1400 m varied techniques	5 min	2 × 5 ER pull OR SWIMWU CONT 2 min	Yes	25-m front crawl Peak and mean thrust Speed	8 min	~ 13.4% increase in peak thrust (~ 9.2 N) post-ER compared with CONT	0.71
									18.9% increase in mean thrust (~ 4 N) post-ER compared with CONT	0.49
									~ 2.8 increase in speed post-ER compared with CONT (0.02 m·s^−1^)	0.20
Bodyweight CA										
Abbes et al. [30]	17 M (13 ± 2y) regional youth swimmers Age 13 ± 2.0 y	No	SWIMWU 1200 m	20 min	SWIMWU + 1 × MAX Press ups in 30 s OR SWIMWU CONT	Yes	50-m freestyle swim time	10 min	No significant improvements	-
Krzysztofik and Wilk [32]	24 M resistance-trained individuals Exp age 24.7 ± 3.1 y Cont age 24.4 ± 2 y Exp BP1RM = 1.24 × BM Cont BP1RM = 1.25 × BM	Yes	5 min cycling with upper-body component at 100 W 70–80RPM, 2 × 10 trunk rotations and side bends, 10 internal and external shoulder rotations, 10 push-ups	U	3 × 5 PLYO push-ups 1 min	Yes	3 × 3 BP (70%1RM)	4, 8, 12 min	~ 7.3% increase in PPO and ~ 5.1% increase in peak bar velocity 4 min post PLYO	0.28 0.40
									~ 3.8% and ~ 5.3% decrease in PPO and peak velocity 12 min post-PLYO	-
Tsolakis et al. [28]	23 (13 M 10 F) elite fencers M BP1RM = 1.01 × BM F BP1RM = 0.62 × BM Age 22.25 ± 4.25 y	Yes	5 min light jogging, 15 s whole body static stretch holds, incremental BP 50–75%1RM)	3 min	3 × 5 PLYO push-ups 1 min	No	BBT (40%1RM)	15 s, 4, 8, 12 min	No significant differences	-
Ulrich and Parstorfer [25]	16 M recreational athletes BP1RM = 1.18 × BM Age 23.1 ± 3.2 y	Yes	5 min cycling (< 100 W at 60–80 rpm), 5 min upper-body DS	8 min	1 × 10 PLYO push-ups	No	BBT (30%)	1, 4, 8, 12, 16 min	~ 4.9% increases in PPO post-PLYO after 8 min	0.30
Sarramian et al. [53]	18 (10 M 8 F) national level swimmers Age 16.0 ± 1.6 y PU3RM = 67.7 ± 10.4 kg	Yes	15 min SWIMWU	Individual rest times	15 min SWIMWU + 15 min SWIMWU CONT OR 15 min SWIMWU + 1 × PU3RM	Yes	50-m freestyle swim time	Individual rest periods	~ 0.9% increase (0.36 s) in swim time following PU3RM compared with SWIMWU CONT	-
Bliss et al. [49]	13 M skilled golfers Age 20 ± 1 y	No	2 min skipping, 1 × 10 leg swings, 2 × 10 ER shoulder external rotations, 1 × 10 kneeling kickbacks, 1 × 10 lunges with rotations, 1 × 12 OH squat, golf-specific shots	U	BW 2 × 10 PLYO press-up + 3 × 10 CMJ	Yes	Clubhead and ball speed, carry distance and total distance	1 min. Then 1 drive per minute for the next 9 min	~ 1.4% and 0.8% increase in clubhead and ball speed, respectively, post-BW trial compared with CONT	0.27 0.17
									~ 2.1% and 0.9% increase in carry and total distance post-BW compared with CONT	0.35 0.15
Combination of exercise CA										
Gelen et al. [47]	26 U elite youth tennis athletes Age 15.1 ± 4.2 y	Yes	TWU; 5 min jogging, 5 min rally, 5 min practice serves	2–4 min	1 × 20 “Ballistic Six” Latex tubing external rotation Latex tubing 90/90 external rotation Overhead soccer ball throw, 90/90 external rotation side throw Deceleration baseball throw, baseball throw 60 s OR 2 × 15 upper-body DYNWU; internal/external rotation with racket supination/pronation with racket, trunk rotation with racket 30 s	Yes	Tennis serve velocity	2–4 min	~ 3.4% increase in serve velocity post-ballistic six compared with TWU	0.70
									~ 2.1% increase in serve velocity post-ballistic six compared with upper-body DYNWU	0.41
									~ 1.3% increase in serve velocity post-upper-body DYNWU compared with TWU	-0.27

*1RM* one repetition maximum, *BBT* barbell bench throw, *BM* body mass, *BP* bench press, *BW* bodyweight, *C* combined, *CA* conditioning activity, *CMJ* counter-movement jumps, *CONC* concentric, *CONT* control, *CP* cable pulley, *DRYWU* dry-land warm-up, *DS* dynamic stretching, *DYNWU* dynamic warm-up, *ECC* eccentric, *ER* elastic resistance, *F* female, *g* Hedges G*, *ISO* isometric, *M* male, *Max* maximum, *MB* medicine ball, *m.s*^*−1*^ m per second, *mins* minutes, *N* Newtons, *NCCA* National Collegiate Athletic Association, *OH* overhead, *OHB* overhead backward throw, *PAPE* post-activation performance enhancement, *PC* power clean, *PLYO* plyometric, *PO* power output, *PPO* peak power output, *pro* professional, *PU3RM* pull-up 3 repetition maximum, *RTH* resistance training history, *SB* standard bat, *SS* SuperSpeed, *SWIMWU* swim warm-up, *SWU* standard warm-up, *TWU* traditional warm-up, *U* unknown, *VR* variable resistance, *WBS* weighted bat swings, *WU* warm-up, *y* years

Seitz and Haff [4] note that this method is preferable in pre-test and post-test design studies in meta-analyses, citing Morris [21] who suggested that this method shows superior properties with respect to bias, precision, and robustness to heterogeneity of variance compared with other methods. The corrected ES was then calculated as follows (Eq. 4):

4 $${\text{Corrected ES}} = g \times {\text{correction factor}}$$

The following thresholds were used: trivial = 0.20, small = 0.20–0.49, moderate = 0.50–0.80, and large =  ≥ 0.80 [22].

### Meta-analysis

Where appropriate, a meta-analysis was performed using RevMan, version 5.4 [23]. The standardised mean difference of pre-post changes was calculated, and heterogeneity between studies was assessed by observing the *I*^2^ statistic, at the following thresholds: 0–40% may not be important, 30–60% may represent moderate heterogeneity, 50–90% may represent substantial heterogeneity and ≥ 75% may represent considerable heterogeneity [24].

## Results

A total of 127 studies pertaining to the search criteria were identified for further analysis, of which 16 duplicates were removed. Titles and abstracts of the 111 manuscripts were initially screened for their relevance, followed by a full-text review of the remaining 57 relevant manuscripts to assess their eligibility in accordance with the inclusion criteria. After analysing eligibility, 31 studies were selected to be included in the review (Fig. 1).

### Risk of Bias Analysis

As identified in Table 2, the methodological quality of 26 studies was ≥ 5/9, whilst five studies were classed as 4/9, which translated to median score of 5. No studies were identified as < 4. Criteria 1 and 6 were omitted because of a lack of relevance. Minimal information for criteria 5, 6 and 7 was evident because of the nature of the experimental studies.

### Characteristics of Participants in the Included Studies

Studies were published from 2005 to 2021 and included a total of 565 individual participants. This comprised 404 male individuals and 101 female individuals, the sex of 60 participants were not provided. The participants were largely of an athlete population (85%), with an equal distribution of professional and amateur/recreational athletes as determined by the participant information disclosed by the study’s authors. The other 15% were defined as resistance trained.

### Bench Press Variation Conditioning Activities

Bench press variations were the most frequently utilised PAPE conditioning activity, with 14 studies including this type of activity. This comprised the bench press, eccentric-only bench press, concentric-only bench press and isometric bench press, in addition to the ballistic bench throw and variable resistance variations. All studies included low-volume protocols, from 1 to 3 sets, of 1–6 repetitions as seen in Table 3. Load varied across each bench press variation and is described in more detail in the following sections. Rest periods between consecutive conditioning sets, where applicable, also varied between studies. This typically ranged from < 1 min to 4 min, whilst one study comprised individualised rest periods [27]. There was also disparity in the rest period from the cessation of the conditioning activity, and the following performance test between studies. Some authors monitored performance at single timepoints, whilst other authors reported data at multiple timepoints to identify the time course of PAPE. Overall, this ranged from 15 s to 24 min post-conditioning activity.

Small-to-moderate (ES = 0.06–1.34) performance increases in ballistic bench throw power output following a bench press variation conditioning activity were reported in seven studies [25, 27, 35, 38, 40, 41, 54]. This translated to a peak improvement ranging from ~ 3.3 to 5.7%. Conversely, two studies reported no significant improvements [28, 34] and one study reported a marginally detrimental effect [35]. An ~ 4.3% improvement in ballistic bench throw was reported 8 min following 3 × 3 bench press at 87%1RM in professional rugby players [27]. Other studies found 8 min to be an optimal recovery duration to elicit PAPE following bench press at similar loads, where several timepoints were considered [25, 38, 41]. No improvements in the throwing velocity of amateur handball players were found following a bench press conditioning activity of 90%1RM [55]. Similarly, no increases in throwing velocity of a light-weight (0.55-kg) medicine ball were observed following several sets of bench press [33], though improvements (~ 8.9%) were observed in the 4-kg medicine ball condition. No improvements were found in bench press power output when preceded by 3 × 3 bench press at 85%1RM [31]. An increase in power output was observed in the concentric-only bench press, 7 min following a single set of bench press at 1RM [29]. Only a single study explored the use of a concentric-only bench press to improve subsequent ballistic bench throw in competitive rugby players [35]. The authors found that a single set of 3 repetitions at 3RM induced a small increase (~ 3.3%; ES = 0.15) in the ballistic bench throw power output. In contrast, concentric-only bench press did not improve subsequent upper-body plyometric performance [39]. No studies showed improvements in performance following an eccentric-only bench press conditioning activity [25, 35]. No improvements in BBT power output were found following an isometric bench press conditioning activity of 3 × 3 s [28]. In contrast, an ~ 2.8%; ES = 0.14 improvement in power output in the BBT was found following 1 repetition of isometric bench press of a 7-s duration [35]. A standing isometric chest press variation did not elicit PAPE in experienced handball players [46], observed as no improvements in overhead handball throwing velocity.

### Sport-Specific Conditioning Activities

The following sub-sections highlight the results from studies where authors used sport-specific conditioning activities, comprising either the sporting action or a conditioning activity that shared biomechanical similarities to the performance test.

#### Modified Implement Throw Conditioning Activities

Three studies found that the use of overweight implement throws, 3 min prior to competition weight throws, elicits a performance enhancement, as observed by an increase in distance (~ 1.7–8.5%; ES = 0.14–0.33) [26, 37, 42]. A single study found that a single set of six bowls of a heavier (+ 10%) cricket ball did not improve accuracy or speed in subsequent cricket bowls, compared to a control trial [50]. Indeed, similar results were reported in the lighter ball (− 10%) condition [50].

#### Swing-Specific Conditioning Activities in Ball Striking Sports

Five studies were included in the review pertaining to ball striking sports [36, 43, 49, 51, 52]. Improvements in golf club and ball speed (~ 1.4%; 0.9%) and carry and total distance (~ 2.6%; 1.4%) were reported following a prior conditioning activity of light and heavy golf-specific implement swings [49]. Likewise, a single study reported an increase (~3.3%) in swing velocity after lighter baseball bat swings, compared with a standard bat [36]. On the contrary, three studies have shown a lack of improvement, and in some cases, a detrimental effect on performance, when prior swings are performed with a heavier bat [36, 51, 52]. Two studies [43, 51] have observed increases in bat swing velocity (~ 1.3–4.9%) following a conditioning activity of isometric bat swings.

#### Cable Pulley Conditioning Activities

Three studies explored the use of a cable pulley-type mechanism as a ‘sport-specific’ PAPE conditioning activity [44, 48, 55]. An improvement in swim time (~ 0.9%) was reported 6 min following a PAPE conditioning activity of 4 × 7 s of resisted swim sprints [44]. One study found improvements in rate of force development (~ 9.4%) and stroke rate in a resisted front crawl swim, following a single set of three cable pullovers [48]. However, the same study reported decreases in several performance variables such as velocity, power, force and acceleration when compared with a control trial. No improvements in handball throwing velocity were reported following eccentric conical pulley activity in amateur handball players [55].

#### Elastic Resistance Conditioning Activities

Moderate improvements in peak (~ 13.4%; ES = 0.71) and mean (18.9%; ES = 0.49) thrust and speed (~ 2.8%; ES 0.20) were observed in international and national-level swimmers following a conditioning activity of elasticated pulls, compared with a control trial [45].

### Bodyweight Conditioning Activities

Six studies included in the review investigated the efficacy of bodyweight PAPE conditioning activities [25, 28, 30, 32, 49, 53]. An ~ 4.9% increase in power output in the ballistic bench throw was reported 8 min following a PAPE conditioning activity of 1 × 10 plyometric push-ups [25]. In contrast, a separate study found that 3 × 5 repetitions of the same conditioning activity failed to elicit performance improvements in the ballistic bench throw [28]. Another study reported improvements in power output and bar velocity in the bench press, following 3 × 5 plyometric push-ups [32]. A PAPE conditioning activity of 2 × 10 plyometric push-ups in addition to 3 × 10 counter-movement jumps elicited increases in clubhead (~ 1.4%; ES = 0.27) and ball speed (0.8%; ES 0.17), and carry (~ 2.1%; ES = 0.35) and total distance (0.9%; ES = 0.15) in skilled golfers, compared with a control trial [49]. The addition of a single set of 3RM pull-ups to a swimming warm-up did not induce performance benefits in 50-m freestyle swim time, compared to a control trial of the swimming warm-up only [53].

### Combination of Exercise Conditioning Activities

A single study asked tennis players to perform a combination of exercises to explore whether such conditioning activity could induce performance improvements [47]. An upper-body dynamic warm-up elicited an ~ 1.3% increase in tennis serve velocity when compared with a traditional warm-up. Further improvements were found compared with the control trial, following a ‘ballistic six’ condition, whereby athletes performed several upper-body rotational ballistic movements.

### Study Results

Table 3 displays the participant and experimental details, and the results of the literature pertaining to upper-body PAPE.

### Meta-analysis

**Fig. 2:**
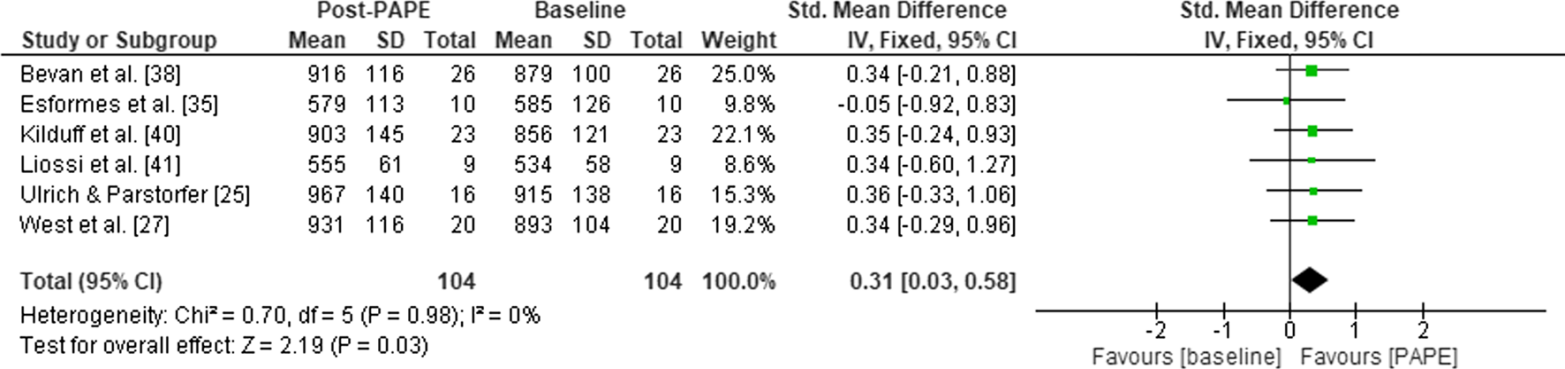
Forest plot for power output in the barbell bench throw pre and post a bench press conditioning activity at ≥ 80%1RM. *CI* confidence interval, *IV* inverse variance, *PAPE* post-activation performance enhancement, *SD* standard deviation, *Std* standard

Because of the variations in the experimental design of the studies (randomised controlled trial and pre-post), a meta-analysis was appropriate for only one subset of studies that included a pre-post-test design; the bench press and ballistic bench throw (BBT) complex (Fig. 2). This was possible because of the similarity in conditioning protocols and outcome measure (power output in the ballistic bench throw).

Six studies investigated the effect of heavy (≥ 80%) bench press on power output in a subsequent BBT [25, 27, 35, 38, 40, 41]. The use of the heavy bench press as a conditioning activity had a positive effect (*p* = 0.03) on BBT performance compared with baseline (ES = 0.31; CI 0.03–0.58) [Fig. 2]. No heterogeneity was observed (*I*^2^ = 0%, *p* = 0.98).

## Discussion

This study aimed to perform a systematic review and meta-analysis on the efficacy of upper-body PAPE conditioning activities. Data suggest that several upper-body conditioning activities can be used to acutely improve upper-body performance. Findings varied between studies, at times because of differences in experimental designs and procedures, as discussed in later sections.

### Bench Press Variation Conditioning Activities

As highlighted in the meta-analysis, the bench press with loads of ≥ 80%1RM can be an effective conditioning activity to elicit improvements in subsequent power output in the BBT at 30–40%1RM [25, 27, 35, 38, 40, 41]. Only one study [35] reported a marginally detrimental effect on BBT power output. Peak power output was typically observed between 8 and 12 min post-conditioning activity; however, this varied because of the different timepoints considered. Where performance at multiple timepoints was considered, three studies reported a peak in BBT power output after 8 min rest [25, 38, 41], whilst one study reported a peak at 12 min [40]. All of the above studies implemented a bench press protocol of ≥ 80%1RM, which could suggest that a heavy load bench press conditioning activity requires a considerable recovery duration to induce PAPE. It should be noted, however, that loads were rarely directly compared in the literature. Liossis et al. [41] found 4 min was optimal recovery following bench press at 65%1RM, whilst 8 min was considered optimal for the 85%1RM load. This again highlights the trade-off between fatigue and PAPE. Similarly, 7 min following a bench press protocol at 90%1RM was sufficient recovery to observe a significant increase in concentric-only bench press power output [29]. Coaches and practitioners may wish to use the bench press as a conditioning activity to evoke PAPE in subsequent BBT; however, as noted by Seitz and Haff [4], greater recovery durations may be needed where high loads are used.

The only study that utilised a heavy bench press protocol on subsequent bench press power output found no significant improvements [31]. However, the 3 × 3 protocol of 85%1RM in that study was described as ‘reps to failure’, which is perhaps not synonymous with producing efforts of maximum power output. Further research is needed to analyse the effects of prior heavy bench press on subsequent bench press power output.

Bench press also failed to elicit improvements in handball throwing velocity [55]. Firstly, the biomechanical differences between the conditioning activity and the performance test are apparent [4], which may suggest the bench press may not transfer to acute task-specific performance improvements. A recent review found the greatest PAPE was typically observed ≥ 5 min following high-to-moderate intensity conditioning activities [4]. It could, therefore, also be plausible that the single measurement at 4 min [55] may not have been sufficient recovery to reduce fatigue from the multiple repetitions of 90%1RM bench press. The same limitations applied to a study by Martinez-Garcia et al. [46], who found no significant improvements in overhead handball throwing velocity following a standing chest press with variable resistance, with even shorter initial recovery durations. Markovic et al. [33] also found bench press produced non-significant differences in throwing performance 3 min following the conditioning activity, where the medicine ball load (0.55-kg) was similar to that of a handball. However, the latter study did find a significant improvement (*p* = 0.001; 8.3%) in throwing distance at the same rest period, with a heavier (4-kg) medicine ball. The bench press may have potential in improving sport-specific performance such as throwing, though this may be load specific [33]. The monitoring of only one timepoint in both studies could mask potential PAPE effects that manifest at greater recovery durations, which may mislead the coach or practitioner on the efficacy of such conditioning activities. However, it is worth noting that several testing intervals may also negatively affect subsequent performance due to fatigue, therefore requiring careful consideration.

Only a single study [35] explored the use of concentric-only bench press as a conditioning activity to improve BBT power output, evidencing potential effectiveness. Similarly, only one study explored the use of concentric-only bench press on subsequent plyometric activity [39], finding it may not be an effective method to acutely improve performance in the plyometric push-up. Post-activation performance enhancement was monitored at only one timepoint following the conditioning activities in the two studies [35, 39], 12 min and 1 min, respectively. Again, it is conceivable that performance improvement could manifest at later timepoints, when considering the time course of PAPE is thought to last for several minutes [4]. In the initial minutes following the conditioning activity, other mechanisms such as PAP may be the basis of performance improvement [5], suggesting the 1 min recovery period in the study by Bodden and colleagues is inappropriate in relation to PAPE. This is especially true when reflecting on the incremental high-volume protocol involved [39]. The lack of studies exploring the efficacy of concentric-only bench press to induce PAPE suggests the evidence is unclear.

The evidence presented in this review suggests that eccentric-only bench press does not induce a PAPE effect in BBT power output [25, 35]. Both studies reported no significant differences as a result of the conditioning activity. Though high loads have been demonstrated to induce PAPE throughout this review, the supramaximal nature (120%1RM) of the conditioning activity in the study by Ulrich and Parstorfer [25] may have induced too much fatigue, thus suppressing PAPE. This is despite the lower number of overall reps (1 × 3) performed compared with bench press PAPE studies. Esformes et al. [35] used a lighter load, equivalent to 3RM, and found only a very marginal increase (0.8%) in power output. Therefore, the PAPE-inducing capability of the eccentric-only bench press on the BBT is not currently supported by the literature.

The two studies that included an isometric variation presented conflicted findings [28, 35]. Tsolakis et al. [28] found no significant differences in BBT power output following isometric bench press. Further analysis revealed that the female athletes possessed relative strength levels (bench press 1RM as a percentage of body mass) of 0.62, noticeably lower than the 1.01 achieved by the male athletes in the same study. As strength level is a modulating factor of the PAPE response [4], the large differences in strength levels could explain the apparent lack of performance improvement. Specifically, stronger athletes are able to achieve greater levels of PAPE (ES = 0.41), compared with their weaker counterparts (ES = 0.32) [4]. It has been suggested that an increased level of strength may make an individual more resistant to fatigue following a conditioning activity, thus responding more favourably than weaker athletes [4].

In contrast, Esformes et al. [35] did identify significant improvements in subsequent BBT power output, where participants possessed overall relative strength levels (bench press 1RM as a percentage of body mass) of 1.1 in the bench press. Additionally, the latter study included only one maximal voluntary contraction, whereas Tsolakis et al. [28] included three. Differing protocols and the ensuing fatigue may contribute to the varied results, though the overall contraction time was similar in both studies. The lack of research makes it difficult to infer the effectiveness of an isometric bench press conditioning activity in inducing an acute performance benefit in the BBT. Efficacy may be contingent on athlete strength levels, and by association, sex.

West et al. [27] reported increases in BBT power output, 8 min following a 3 × 3 BBT conditioning activity at 30%1RM. In contrast, separate trials of 1 × 5 BBT at various loads ≥ 50%1RM did not influence BBT power output after 4 min rest [34]. The disparity between the two studies may be explained by the increased recovery time in the former study. A single set of 3 repetitions of BBT at 30% did not elicit any differences in subsequent BBT performance [54]. Interestingly, in the same study, a contrast protocol of 1 × 3 BBT at 30%1RM, followed by 1 × 5 at 60%1RM load did produce an improvement in power output at 3 min (8.7%) and 5 min (10.4%), respectively. The differences at the two timepoints again support the notion of optimal post-intervention timing. The evidence suggests that the BBT as part of a resistance exercise contrast or complex set may be a promising conditioning method to induce PAPE, and thus, improve subsequent performance in the same activity. However, as is a common theme throughout, more research is required to confirm this.

Further research could explore whether bench press variations can acutely influence task-specific or sporting performance. This would enable practitioners and coaches to determine whether it has appropriate inclusion as a pre-competition warm-up conditioning activity for athletes. Though, as previously discussed, biomechanical specificity and logistical issues mean more sport-specific PAPE conditioning activities could be better alternatives for athletes.

### Sport-Specific Conditioning Activities

Select studies explored the use of movement-specific combinations that involve the sporting action as the conditioning activity and performance test, thus greater specificity to sporting performance. These complexes can be used more widely for athletes outside of a weight room or laboratory, for example, within the constraints of the competition warm-up environment and are described in the sections below.

#### Modified Implement Throw Conditioning Activities

The current data suggest that warming up with overweight implements could potentially improve subsequent throwing for distance performance; however, the previous literature suggests there may be a limit to performance benefits at increased loads [26, 37, 42]. A plausible reason for the improvement could be due to the conditioning activities and the performance tests being either identical or at least sharing biomechanical similarities [4]. It is worth noting that although the participants were trained in their respective throwing disciplines, strength levels in the studies varied [26, 37], whilst one study did not report this information [42]. All studies shared a 3 min recovery period between the cessation of the conditioning activity, and the performance measure. Whilst this suggests that a 3 min period may be sufficient to elicit a performance improvement following a throwing conditioning activity, the time course of PAPE is purported to last several minutes [4]. Thus, the lack of monitoring at several timepoints may underestimate a ‘peak’ in the PAPE effect. Though, it is again worth noting the potential negative performance effects that could arise from excessive testing intervals. Nevertheless, on current evidence, warm-up throws with a marginally overweight implement compared with the competition standard seem to be an effective method of improving competition throwing performance where increased distance is the primary goal. Therefore, throwing coaches could consider adopting this well-practiced strategy either as a pre-competition conditioning activity or in-between throws in training, once individual athlete responsiveness has been established. In contrast, the use of a heavy cricket ball, and indeed a lighter cricket ball, did not yield any performance improvement in cricket bowling speed and accuracy [50]. Again, the latter study included a short recovery duration of 3 min between the conditioning activity and the subsequent performance test. Likewise, participant strength levels were not reported.

#### Swing-Specific Conditioning Activities in Ball Striking Sports

Five studies were included in the review pertaining to ball striking sports [36,43,49,51,52]. Bliss et al. [49] reported improvements in speed and distance in the golf swing, 1 min following prior swings with light and heavy implements, compared with a control trial. The study by Bliss et al. [49], and indeed all of the swing-specific sports included in the review, comprised PAPE conditioning activities that shared clear biomechanical specificity to the performance test in terms of the movement pattern. Bat swing-specific isometric contractions of a 5-s duration seemingly induce a PAPE effect on subsequent swing velocity [43, 51]. Gilmore et al. [43] demonstrated a PAPE effect following 3 × 5 bat swing-specific isometric contractions, in a group of athletes that are not considered to be the strongest, whereas Higuchi et al. [51] did not report strength characteristics. Interestingly, the latter study showed an ~ 1.3% increase in swing velocity after just 1 min of rest post-conditioning activity. The evidence on the efficacy of weighted swings would suggest it does not improve, and in some cases, may even be detrimental to subsequent swing velocity [36, 51, 52]. Where Bliss et al. [49] reported improvements post ‘heavy’ swings, this was actually of a similar mass to that of a standard golf club, and was part of a protocol of contrasting implements. Montoya et al. [36] reported an increase (3.3%) in swing velocity after lighter bat swings, compared with a standard bat. All studies pertaining to swing-specific conditioning activities in ball striking sports had administered the initial post-performance test within 1 min of the conditioning activity. Considering the proposed time course of PAPE and the possible coexistence of PAP and PAPE [5, 16], any performance improvements may not necessarily be solely attributed to PAPE. Despite the small body of work in the area, the use of bat swing-specific conditioning activities in the form of isometric contractions appear to be a useful method for improving subsequent swing velocity. This method has been applied in baseball and softball, though it may offer a novel method for the coach and practitioner across other sports that require upper body distal point velocity, such as throwing or punching. The use of lighter weighted swings or contrasting light-heavy swings may be useful in improving subsequent swing-specific performance [36, 49]; however, more research should explore the efficacy of this method.

#### Cable Pulley Conditioning Activities

Three studies explored the use of a cable pulley-type mechanism as a ‘sport-specific’ PAPE conditioning activity. Asencio et al. [55] did not find any improvements in handball throwing velocity after eccentric conical pulley activity, with the authors stating this was possibly due to the short recovery time of 4 min. Hancock et al. [44] implemented a longer recovery duration of 6 min following the swimming-specific mode and found an ~ 0.8% reduction in swim sprint time, corresponding to just under a 1-s improvement. Cuenca-Fernandez et al. [48] also administered a 6 min recovery period to national-level swimmers post-dynamic stretching and 1 × 3 cable pull overs at 85% of 1RM. Compared with a control trial of a swimming-only warm-up, rate of force development and stroke rate improved in the 15-m front crawl. Interestingly, the conditioning activity produced a negative effect on variables such as velocity and distance covered. More research is needed to understand the potential benefits of the cable pulley or similar resistance, as a PAPE-inducing conditioning activity.

#### Elastic Resistance Conditioning Activities

Only one study was included in the review that explored the efficacy of a PAPE conditioning activity with elastic resistance [45]. Increases in the thrust and speed of international and national-level swimmers’ 25-m front crawl performance was reported following 2 × 5 elasticated pulls performed 8 min prior. The inclusion of only one study does perhaps show an under-utilisation of elastic resistance to induce PAPE. Elastic resistance has successfully induced a PAPE effect in combat-specific actions [56, 57]; however, because of the lower-body elements of the conditioning activities, the relevant studies were not included in this review. Therefore, future research should further explore the efficacy of upper-body elastic resistance activity in inducing PAPE in a variety of sports.

### Bodyweight Conditioning Activities

Sarramian et al. [53] compared the effects of a swimming warm-up inclusive of 1 × 3 pull-ups at 3RM, to a control trial of just a swimming warm-up. The findings showed the addition of the pull-up conditioning activity did not improve 50-m freestyle swim performance to a greater extent than the swimming warm-up, despite the inclusion of individual rest times. The only study to explore the PAPE-inducing effect of the push-up, included a 30-m freestyle swim as the performance test [30]. The authors found no significant improvements compared to a control trial of rest. A number of factors could explain the lack of performance improvement. Firstly, the protocol was reported as a 30-s maximal effort. This conditioning activity may induce levels of fatigue that would suppress any possible PAPE effect. Indeed, the authors reported mean pre-swim blood lactate levels of 3.6 ± 0.9 mmol.l^−1^ during the push-up trial, compared with 1.9 ± 8 mmol.l^−1^ at the same point in the control. There is also arguably little biomechanical specificity between the push-up and freestyle swim technique; however, it should be noted that it is not a requirement for the conditioning activity to directly mimic the sporting activity [11]. Lastly, no information on the strength levels of participants was provided by the authors.

Plyometric versions of the push-up have received more focus in the literature. Ulrich and Parstorfer [25] found the plyometric push-up to be an effective conditioning activity to improve subsequent BBT power output by ~ 4.9% after 8 min. Both exercises share similar stretch–shortening cycle principles, whereby a rapid eccentric loading is followed by a rapid concentric push [58]. This again perhaps highlights the importance of biomechanical specificity. Other authors [28] reported only a marginal and non-significant increase in BBT performance, when preceded by a plyometric push-up protocol, comprising the same recovery time implemented by Ulrich and Parstorfer [25]. However, the former study included both female and male participants, with female participants exhibiting considerably lower strength levels, resulting in markedly greater strength levels in the study by Ulrich and Parstorfer [25]. Krzystofik and Wilk [32] found increases in peak power output and bar velocity 4 min post 3 × 5 plyometric push-ups compared with a control trial; however, both variables were weaker than the control trial at 12 min post. Krzystofik and Wilk [32] also reported considerably greater strength levels in their study, compared with that of Tsolakis et al. [28]. As PAPE seemingly manifests in a greater magnitude in stronger athletes [4], this could partly explain the different magnitudes of PAPE observed between the studies. Additionally, Tsolakis et al. [28] instructed participants to complete 15 overall repetitions compared with ten in the study by Ulrich and Parstorfer [25]. Plyometric push-ups performed alongside counter-movement jumps elicited small increases in golf drive speed and distance compared with a control trial [49]. The inclusion of the lower body element, however, makes it difficult to attribute the performance increase to the plyometric push-up.

In summary, it is not yet possible to infer the usefulness of push-ups as a conditioning activity to induce PAPE. However, the findings of three studies [25, 28, 32] suggest that the plyometric variation of the push-up may have potential as a conditioning activity to induce PAPE in the bench press, BBT or golf drive performance.

### Combination of Exercise Conditioning Activities

One study included in the review was categorised on its own, due to the combination of many types of activity [47]. Gelen et al. [47] found increases in tennis serve velocity following an upper-body dynamic warm-up, inclusive of movements with the tennis racket. Interestingly, even greater benefits were found following a ‘ballistic 6’ protocol, whereby the tennis athletes performed a combination of upper-body rotational movements with elastic resistance and implement throws. This suggests a conditioning activity inclusive of ballistic movement may induce PAPE, as seen in the previously discussed sport-specific sections. It is, however, difficult to infer which activities are responsible for the performance improvements.

## Limitations

This review has demonstrated the potential of many upper-body exercises in inducing PAPE, thus improving subsequent athletic performance. The following sections briefly summarise the limitations of the included studies and that of the current review, with accompanying recommendations on potential future research direction and design.

### Limitations of the Included Studies

Whilst PAPE and fatigue can coexist [59], the magnitude of the two can be dependent on many factors, including but not limited to, the type of conditioning activity and the recovery duration. Unfortunately, the body of literature on upper-body PAPE has typically consisted of varied conditioning activity and performance test complexes, in addition to a range of recovery durations, making it difficult to directly compare findings. Researchers should ensure an appropriate time course is applied in monitoring the response to a conditioning activity, particularly considering PAPE could last for several minutes [4, 5]. This would also allow for the analysis of individual PAPE responses [13], whereby an optimal recovery duration could be implemented for individual athletes. The reporting of group and individual characteristics such as age, strength level and training history, to name a few, would allow for a greater analysis of the relationship between these factors, and the PAPE response in responders and non-responders.

An element that has often been ignored in the pre-post study designs is the effect of the warm-up on the apparent PAPE effect. The many benefits of a warm-up to athletic performance has been established previously [1–3]. If it is currently accepted that the mechanisms of warm-up and PAPE are similar [5], then it is difficult to isolate the two elements and correctly attribute a performance enhancement in pre-post study designs, in the absence of a control trial. There are several examples in the literature where details of the warm-up or the recovery durations between the warm-up, conditioning activity and baseline tests were scarce. Future studies should include a pure control trial inclusive of a standardised warm-up and an experimental trial inclusive of a standardised warm-up and conditioning activity, so that potential performance improvements that may be observed can be attributed to the conditioning activity, and not confounding variables [5]. An additional important consideration for the warm-up that was neglected in many studies was a lack of sport or ‘task-specific’ activity, which Blazevich and Babault [5] note may invalidate the real-world applicability of the warm-up.

The current review revealed 11 studies did not include details on familiarisation trials, whereby participants initially become accustomed to the conditioning activities and performance tests to be included in the experimental trials to minimise potential learning effects [60, 61]. Lastly, whilst research exploring the effectiveness of upper-body conditioning activities on isolated sporting or exercise tasks exists, there is seemingly a lack of research exploring the PAPE effects on continuous competitive or simulated match play. For a deeper understanding of how PAPE can impact athletic performance, future research could consider monitoring the effects of PAPE on performance to competitive and simulated situations.

### Limitations of the Current Review

The majority of studies in the bench press and BBT complex meta-analysis comprised pre-post observational designs; therefore, the pre-post standardised mean difference of studies could be calculated for the meta-analysis. The issues of pre-post-test standardised mean difference to indicate treatment effects in meta-analyses have been highlighted in recent research, primarily because of an increased risk of biased outcomes [62]. This can result in less reliable information being calculated about the effects of an intervention, as it only calculates the change within one group. Specifically, pre-post-test designs are not independent of each other, and can be influenced by factors external to the intervention [62]. Notwithstanding the above issues, the authors appreciate that this type of analysis may still be considered useful to the coach and practitioner in the absence of randomised controlled trials, providing the data are interpreted with caution. Additionally, because of the large variations in the methodological approach between studies, meta-analyses of the other conditioning activity classifications were not possible. Future research must aim for greater uniformity in its methodologies. This would assist in the easier interpretation of findings for the coach, practitioner and the meta-analyst.

## Conclusions and Practical Applications

The current review highlights several upper-body conditioning activities that can potentially induce a PAPE effect on athletic performance. Bench press was the most frequently used conditioning activity, proving an effective method to induce a 3.9–5.7% increase in BBT power output following 8–12 min recovery, where bench press loads were between 80 and 87%1RM. This may offer the coach and practitioner a useful complex to improve athlete upper-body strength and power. The eccentric bench press, however, seemingly does not improve subsequent BBT performance, and may indeed impair performance when prescribed at supramaximal loads. Further research is needed to identify the efficacy of other bench press variations such as concentric only, isometric, and ballistic on subsequent ballistic, plyometric and throwing performance, as the literature is currently conflicted. This is important for the coach and practitioner, as understanding whether bench press variations transfer to improved ‘sport-specific’ movements may influence potential warm-up or resistance training complex prescription, in non-powerlifting athletes.

This review supports the prescription of overweight implement throws, prior to competition throw performance where the aim is for maximum distance. Coaches and practitioners could utilise this method in the pre-competition warm-up with throwing athletes, though they should consider whether there will inherently be a weight limit, whereby an increase in the overweight throw implement past a specific threshold may yield diminishing returns. Likewise, this review found movement-specific bat swing activity, in the form of lighter-weighted bat swings and isometric swings, could be promising strategies to acutely improve swing velocity. This strategy could perhaps improve distal point velocity, which could extend the application to striking sports such as boxing, whereby peak fist velocities is an important factor in effective punching.

Other movement-specific complexes that could have potential include resisted activity with a cable pulley type mechanism, select bodyweight activity and elastic resistance, though there is currently either conflicting results or a lack of literature on these conditioning activities. When volume is kept relatively low, this review also highlights the potential efficacy of the plyometric push-up, in improving subsequent ballistic performance in well-trained athletes.

Movement-specific combinations appear to be more successful in producing a performance improvement, in agreement with recent reviews on whole-body acute PAPE [4, 16]. The coach and practitioner may wish to utilise some of the upper-body conditioning activities highlighted in this current review. Coaches and practitioners should, however, interpret the data with caution, owing to the many limitations in the previous literature that are highlighted throughout. To combat this, the authors have provided a very brief guidance on how future PAPE research could be improved. It is hoped this may assist in a greater uniformity in the methodological approach, a wider appreciation of potentially effective exercise modes outside of powerlifting techniques, and thus, a greater understanding of PAPE efficacy for coaches and practitioners.

## Declarations

### Availability of data and material

The datasets analysed during the current study are available from the corresponding author on reasonable request.
