# Correction to: Upper-Body Post-activation Performance Enhancement for Athletic Performance: A Systematic Review with Meta-analysis and Recommendations for Future Research

**DOI:** 10.1007/s40279-021-01623-6

**Published:** 2021-12-17

**Authors:** Mitchell James Finlay, Craig Alan Bridge, Matt Greig, Richard Michael Page

**Affiliations:** Sports Injuries Research Group, Department of Sport and Physical Activity, Edge Hill University, St. Helens Road, Ormskirk, L39 4QP Lancashire UK

## Correction to: Sports Medicine 10.1007/s40279-021-01598-4

**Abstract, Results, final sentence:** This sentence, which previously read:

“Sport-specific lighter weighted bat swings and swing-specific isometrics resulted in improved subsequent competition weight bat swing velocities, ranging from ~ 1.3–3.3%; ES = 0.16–0.57.”

should read:

“Sport-specific lighter weighted bat swings and swing-specific isometrics resulted in improved subsequent competition weight bat swing velocities, ranging from ~ 1.3–4.9%; ES = 0.16–0.57.”

The original article has been corrected.

